# Research and teaching activity in UK occupational physicians

**DOI:** 10.1093/occmed/kqz132

**Published:** 2019-10-23

**Authors:** D. Lalloo, E. Demou, N. Pahl, E. B. Macdonald

**Affiliations:** 1 Healthy Working Lives Group, Institute of Health and Wellbeing, College of Medical, Veterinary and Life Sciences, University of Glasgow, G12 8RZ Glasgow, UK; 2 MRC/CSO Social and Public Health Sciences Unit, Institute of Health and Wellbeing, College of Medical, Veterinary and Life Sciences, University of Glasgow, G2 3QB Glasgow, UK; 3 Society of Occupational Medicine, 20 Little Britain, London EC1A 7DH, UK

**Keywords:** Academic, occupational health, occupational physician, research, teaching

## Abstract

**Background:**

For all doctors, including occupational physicians (OPs), research and teaching are considered core requirements of medical education and continuing professional development. Academic skills are also vital to evidence-based practice and advancement of occupational health (OH) as a specialty. In recent years, attention has focussed on the declining UK OH academic base and the research–practice gap, and increased practitioner participation in research is encouraged.

**Aims:**

To establish a baseline of research and teaching activity among UK OPs, identify related barriers and inform strategies to overcome them.

**Methods:**

An online survey including specific career profile questions derived from consensus following expert panel discussions. It formed part of a larger Delphi study on UK OH research priorities.

**Results:**

We received 213 responses, about 18% of 1207 practising UK OPs. Of these, 162 (76%) undertook research at some career-point, of which 44 (27%) were currently research-active. Similarly, 154 (72%) undertook teaching at some career-point, of which 99 (64%) were currently teaching-active. Of those who had never undertaken research (*n* = 51) or teaching (*n* = 59), 40 and 42% were interested in doing so, respectively. Key barriers were lack of time and opportunity, the former particularly for respondents practising in industry, where ‘commercial’ demands take priority, rather than healthcare.

**Conclusions:**

This study establishes a benchmark of academic activity among UK OPs and identifies related barriers. These ‘target’ barriers can shape research funding priorities and education to increase participation and develop the UK OH academic base.

## Introduction

General Medical Council guidance affirms the expectation that doctors engage in research and teaching throughout their careers [1]. Research-oriented doctors are more likely to demonstrate high-quality care [2]. For occupational physicians (OPs), research and teaching are fundamental to evidence-based practice, professional development [3] and advancement of the specialty, to support the workforce, industry and the economy as workplace hazards become more complex and scientifically demanding [4].

Attention has focussed on the research–practice gap [5], with increased practitioner collaboration encouraged [5]. OPs are well placed to collaborate in research, potentially the first to recognize and highlight newly emerging risks [6].

A declining occupational health (OH) academic base is recognized [3,4,7]. In 2011, approximately seven specialist UK OPs held substantive academic appointments [6]. Now there are no full-time posts and fewer than three full-time equivalents (from OH Academic Forum). Lack of funding and a predominance of OH practice in ‘business-focused’ industry rather than healthcare have been identified as hindrances to research participation [4,7], compared to other medical specialties. Corporate data sensitivity, inadequate industry support, lack of academic experience and perceptions of workplace-health being a secondary priority are contributing factors [3,4,7].c

To build OH research and teaching capacity, an understanding of the current landscape of OP academic activity and related attitudes is imperative. This study aims to establish a baseline of research and teaching activity and attitudes among UK OPs.

## Methods

**Figure 1:**
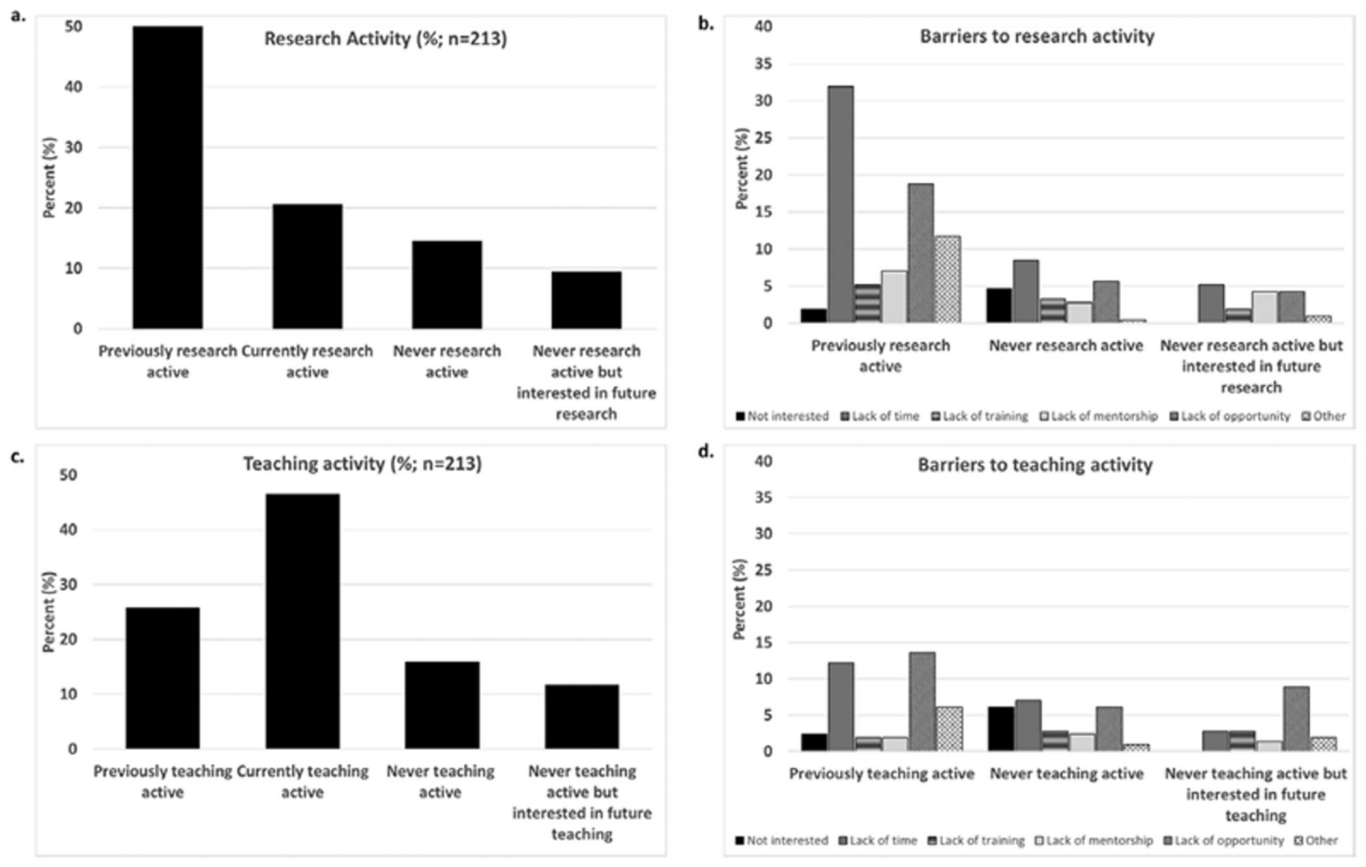
Research and teaching activity: status and barriers.

We conducted an online survey between September and November 2016, as part of a larger study on UK OH research priorities [8]. The detailed methodology is described elsewhere [8]. We developed a first-round Delphi questionnaire from consensus following Faculty of Occupational Medicine (FOM) expert panel discussions (comprising senior OPs). Survey questions included demographics, qualifications, career profile, FOM dissertation experience, past/current teaching/research activity and related attitudes. Respondents selected from a list, a definition that best described their research and teaching activity (Figure 1). We used Excel and R for data analysis. Glasgow University Ethics Committee (200150143) provided ethics approval.

## Results

We received 213 responses (Table 1), about 18% of 1207 practising OPs identified in the FOM Annual report/2017 accounts. Some OPs worked across more than one practice area. In total, 162 (76%) under-took research at some career-stage, with 44 (27%) currently research-active (Figure 1). Of the 51 who were ‘never research-active’, 40% were interested in undertaking research. Lack of time and opportunity were the commonest reported reasons for research inactivity.

Research activity was significantly higher in healthcare OPs (18/63; 29%) compared to industry OPs (12/102; 12%) (*P* < 0.01). Lack of time (58% versus 38%; *P* < 0.05) was a significantly higher barrier in industry than healthcare. Lack of opportunity (33% versus 25%; *P* = 0.37) and training (15% versus 8%; *P* = 0.29) were not statistically different between industry and healthcare. MFOM (Member of the Faculty of Occupational Medicine) and FFOM (Fellow of the Faculty of Occupational Medicine) qualified OPs were significantly more research-active than all other qualified OPs (*P* < 0.001).

Among respondents, 123 (58%) had no research publications; 70 (33%) and 20 (9%) had one to five and six or more publications, respectively. Seventy-three respondents had a Masters degree, 14 a PhD and 7 had both qualifications. For those with Masters and PhDs, 18/73 (25%) and 6/14 (43%) were currently research-active respectively. Of those with PhDs, 4/14 (29%) had no publications; 35 and 36% had one to five and six or more publications, respectively.

Among respondents, 154 (72%) undertook teaching at some career-point; 77 in postgraduate teaching, 13 undergraduate and 64 both; with 99 (64%) currently teaching-active (Figure 1). Of the 59 ‘never teaching-active’, 42% were interested in teaching. Lack of opportunity and time were the commonest reasons for teaching inactivity.

One hundred and thirty-six (64%) had under-taken FOM dissertations, but 53 (39%) reported ‘obstacles’ including lack of statistical and supervisor support, research experience and ethics application. One respondent said:

‘It was difficult. It felt like being told to drive to Glasgow from London, along minor roads, without a map, never having been there before and after just passing your driving test’.

Another highlighted,

‘Ethics committees do not understand OH practice differences and are not valid for non-NHS research’.

However, 120 (88%) considered it ‘time well-spent’ and a ‘useful skill’. Ninety-one (67%) disseminated their results at conferences/meetings, and 53 (39%) published their dissertation. Barriers included lack of support for publishing or not considering it. Some expressed regret at not publishing, as they considered their research relevant to practice.

## Discussion

We present a benchmark of OP academic activity. While approximately three-quarters of respondents undertook research or teaching at some career-point, the present position is reduced. Reported barriers included lack of time, opportunity, previous research experience, supervisor, ethics and statistical support. Reported sector differences identified higher research activity in healthcare and more barriers in industry.

The diversity of career levels and industry sectors of respondents is a strength of our study. Potential biases were mitigated by piloting and expert panel use in questionnaire development. The low response rate limits the generalizability of the results, a limitation reported for other internet-based clinician surveys [9].

Our results support concerns about the declining UK OH academic base [3,4,7]. The sub-optimal research participation of OPs with Masters/PhDs may be attributable to ill-defined academic career-pathways. Some of the identified barriers, such as ethics and publication support, may be readily amenable through educational interventions including online training modules or bespoke courses, consolidated by mentorship from researcher volunteers.

Public and private sector OH providers, as key ‘consumers’ of OH research, should support sustainable development of the OH academic base by encouraging research in their organizations and resourcing time and funding for staff to undertake research and teaching. As co-consumers, employers, including human resources, should promote the research agenda. So should governments, given the wider public health and economic implications of workforce-health [10]. Collaboration among UK OH researchers and centres is vital. This is being addressed through the OH Academic Forum, which is promoting development of a National OH Academic centre. A key objective of this should be to highlight to UK research funders the value and public health impact of OH research.

## Figures and Tables

**Table 1:** Responses by age, sex, country, job title, OH qualification and years of experience

Features	Round 1 (*n* = 213)^a^
Age range category	Frequency, *N* (%)
18–24	0 (0)
25–34	2 (1)
35–44	39 (18)
45–54	72 (34)
55–64	74 (35)
65–74	26 (12)
Total	213 (100)
Sex	
Male	142 (67)
Female	69 (32)
Missing/prefer not to answer	2 (1)
Total	213 (100)
Countries	
England	160 (75)
Northern Ireland	9 (4)
Scotland	31 (14)
Wales	12 (6)
Missing	1 (1)
Total	213 (100)
Job title	
Consultant OP	148 (69)
OP	46 (22)
StR	12 (6)
GP	7 (3)
Total	213 (100)
Clinical practice area	
Industry	102
Healthcare^b^	63
Other	63
Research practice area	
Academic	12
Honorary academic	17
Total	257^c^
OH qualification	
FFOM	74 (35)
MFOM	76 (36)
AFOM	24 (11)
DOccMed	30 (14)
Other	5 (2)
StR in training	4 (2)
Total	213 (100)
Years of experience	Mean ± SD (min-max) *n* = 213 20.1 ± 10.9 (0.5–50)

StR, specialty registrar.

a Approximate response rate of 18% based on FOM annual report/2017 accounts (1207 practising OPs): http://www.fom.ac.uk/wp-content/uploads/AR_2017_FINAL.pdf.

b Healthcare refers to OPs working for the National Health Service (NHS).

c Note that total answers for practice area amount to 257 due to individuals working across more than one area.

## Key learning points

### What is already known about this subject

- For OPs, research and teaching are fundamental to evidence-based practice, professional development and advancement of the specialty and yet a declining OH academic base is recognized.
- To build OH research and teaching capacity, an understanding of the current landscape of OPs' academic activity and related attitudes is imperative.

### What this study adds

- We present a benchmark of UK OP academic activity.
- While approximately three-quarters of respondents were research or teaching-active at some career-point, the present position is reduced with barriers described.
- Sector differences identified higher research activity in healthcare compared to industry and sub-optimal research participation of OPs with Masters/PhDs was observed.

### What impact this may have on practice or policy

- The barriers identified in this study can shape research funding priorities, educational and training programmes and tailored support from key stakeholders to increase participation and thereby develop and grow the UK OH academic base.
